# RNA Sequencing Demonstrates That Circular RNA Regulates Avian Influenza Virus Replication in Human Cells

**DOI:** 10.3390/ijms23179901

**Published:** 2022-08-31

**Authors:** Jie Min, Ying Cao, Haizhou Liu, Di Liu, Wenjun Liu, Jing Li

**Affiliations:** 1 CAS Key Laboratory of Pathogenic Microbiology and Immunology, Institute of Microbiology, Chinese Academy of Sciences, Beijing 100101, China; 2 Savaid Medical School, University of Chinese Academy of Sciences, Beijing 100039, China; 3 National Virus Resource Center, Chinese Academy of Sciences, Wuhan 430071, China; 4 State Key Laboratory for Conservation and Utilization of Subtropical Agro-Bioresources & Laboratory of Animal Infectious Diseases, College of Animal Sciences and Veterinary Medicine, Guangxi University, Nanning 530004, China; 5 CAS Key Laboratory of Special Pathogens, Wuhan Institute of Virology, Center for Biosafety Mega-Science, Chinese Academy of Sciences, Wuhan 430071, China

**Keywords:** Circular RNA, avian influenza virus, human cells, RNA sequencing, replication

## Abstract

Circular RNAs (circRNAs) are involved in diverse biological processes. Avian influenza virus (AIV) can cross the species barrier to infect humans. Here, we employed RNA sequencing technology to profile circRNA, microRNA, and mRNA expression in human lung carcinoma cells in response to AIV or human influenza A virus (IAV) infection at viral replication. The analysis revealed that the expression of 475 common circRNAs were significantly regulated. The 381 and 1163 up-regulated circRNAs were induced by AIV at 8 and 16 h, respectively. Subsequently, gene ontology and Kyoto Encyclopedia of Genes and Genomes analyses were also conducted for the AIV-specific up-regulated circRNAs. Moreover, the circRNAs were characterized, of which six were verified by quantitative real-time PCR. We further confirmed that expression of the selected circRNAs only increased following AIV infection. Knocking down the selected circRNAs promoted AIV proliferation, and overexpression of three of the candidate circRNAs restricted AIV replication and proliferation. We further analyzed that AIV-specific up-regulated circRNA mechanisms might function through the ceRNA network to affect the “Endocytosis” pathway and the “Cell cycle process”. These data provide the first expression profile of AIV-specific up-regulated circRNAs and shed new light on the pathogenesis of AIV infection. Our findings also suggest that these circRNAs serve an important role in AIV infection.

## 1. Introduction

IAV belongs to the *Orthomyxoviridae* family, enveloping eight negative-sense single-stranded RNAs. IAV continues to pose a serious threat to public health, causing hundreds of thousands of deaths each year [1]. In general, IAV has a wide range of species specificity, including humans, birds, and other mammalian sources. It has previously been reported that some viral strains from poultry or pigs can cross the species barrier to infect humans [2,3]. Since 2013, HPAIV H7N9 has caused multiple consecutive human infections with a fatality rate of up to 40% [4]. Therefore, there is concern that the continued evolution of AIV could eventually lead to sustained human-to-human transmission. Among the LPAIV, the H9N2 subtype is the most widespread and destructive. It is considered to be the major donor of the IAV genes and can recombine with co-circulating IAVs to cause zoonosis [5]. In recent years, H9N2 AIV was found to be the gene donor for H7N9 AIV [6]. However, the mechanism by which it mediates an abnormal host response remains unclear.

It is well known that host cells infected with viruses usually mount potent and varied antiviral responses to limit virus proliferation. A large number of host proteins act as interferon-stimulated genes (ISGs) [7] or host restriction factors [8] to inhibit IAV replication. In contrast, to counter host responses, IAV has developed strategies to circumvent innate immunity by utilizing the viral NS1 protein as an interferon-induced antagonist [9], or host factors to promote viral replication. Unlike cellular proteins, the combined role of noncoding RNAs (ncRNAs) in IAV replication remains unclear, though some long ncRNAs (lncRNAs) and miRNAs have recently been reported to be involved in cellular responses during IAV infection. Identifying these ncRNAs and their mechanisms may provide new strategies for suppressing IAV infection.

Based on a 200 nt length cutoff, ncRNAs are divided into short ncRNAs and lncRNAs. miRNAs are a class of ~22 nt ncRNAs that are involved in different biological processes through post-transcriptional regulation at the gene level via mRNA degradation or translational inhibition [10]. circRNAs, newly recognized members of the lncRNAs, are generated by reverse splicing between 3′ and 5′ ends. At present, dozens of host ncRNAs have been shown to play important roles in the pathogenesis of IAV, including lncRNAs, miRNAs, and circRNAs. For example, our previous studies demonstrated that IAV-induced lnc-MxA restricts Interferon-β (IFN-β) transcription by interfering with IRF3 and P65 on IFN-β promoters [11]. Furthermore, we verified that IAV-induced lnc-ISG20 takes part in the post-transcriptional regulation of *ISG20* by acting as a competing endogenous RNA (ceRNA) of ISG20 mRNA by sponging miR-326 to release ISG20 mRNA [12]. Recently, the circRNA AIVR was reported to act as a miR-330-3p sponge, releasing CREBBP mRNA and, thus, accelerating IFN-β production, thereby antagonizing IAV proliferation [13]. In addition, it has been reported that miRNAs can directly target influenza viral mRNA to inhibit IAV replication [14,15] or indirectly mediate the antiviral response by targeting host genes [16,17]. However, no comprehensive analysis of circRNAs, miRNAs, and mRNAs that are expressed in human lung carcinoma cells (A549) infected with different human or avian typical influenza viruses and with high or low pathogenicity IAV, has been reported.

It is well known that AIV does not massively replicate when it infects mammalian cells, usually due to amino acid residues at site 627 of the viral PB2 protein. Interestingly, we previously found that all H7N9 strains used in our experiments have the same poultry residue at sites 627E and 701D of PB2, regardless of whether they are predicted to have a high or low probability of infection in humans [18]. Similarly, a recent study reported that circRNAs activate RNase L and are degraded globally in response to viral infection [19]. Therefore, we wondered whether AIV replication after infecting mammals might increase by hijacking host circRNAs, even if they do not have widely accepted mammalian adaptive mutations. Alternatively, host cells can construct the host barrier by specifically upregulating circRNA through AIV. In recent years, an increasing number of studies have reported circRNAs that were discovered by influenza virus regulatory RNA sequencing. For example, the expression profile of circRNAs and up-regulated circRNA_0082633 in PR8-infected A549 cells inhibit IAV infection by promoting the type I IFN signaling pathway [20]. Similarly, circRNA expression profiles have been reported in chicken DF-1 cells infected with H5N1 virus [21] and in the lung of mice infected with H7N9 virus [22]. circRNA production in MDCK cells infected with canine influenza viruses H3N2 and H5N1 has also been established [23]. However, compared with human IAV, H7N9- and H9N2-specific circRNAs have not been studied.

In this study, we performed global pre-processing of circRNA, miRNA, and mRNA expression in A549 cells infected with three subtypes of human or avian IAVs at two early time points of multicycle replication kinetics. Cis- and trans-analyses were performed by ceRNA network exploration to study the commonalities and differences of their pathogenicity and explore the molecular regulatory mechanisms of circRNAs. Furthermore, real-time quantitative polymerase chain reaction (RT-qPCR) was used to detect whether the expression of six avian-specific up-regulated circRNAs was consistent with the results of RNA sequencing. We then verified their effect on H9N2 replication by knockdown and overexpression of circRNAs. Our study focused on whether circRNAs may be involved in the pre-and post-transcriptional regulation of antiviral response genes through the uptake of miRNAs, which may help guide novel antiviral strategies against influenza virus infection.

## 2. Results

### 2.1. AIV Has Low Replication Ability in A549 Cells

**Figure 1:**
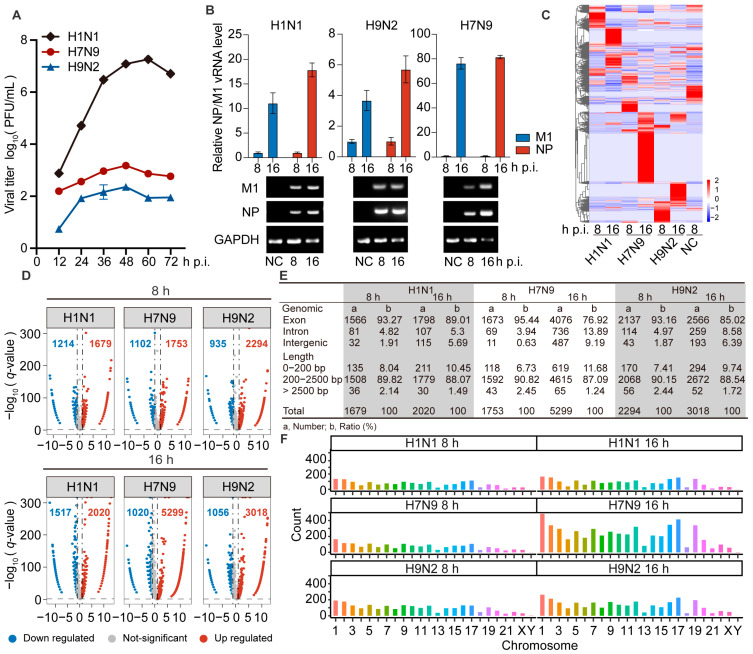
The circRNAs of A549 cells infected with H1N1/H7N9/H9N2 subtype IAV, and uninfected cells were analyzed by RNA-Seq. (**A**) Growth curves of H1N1/H7N9/H9N2 subtype IAV in A549 cells infected at an MOI of 0.001 for 72 h. The virus titer was monitored by plaque formation at the indicated time points (*n* = 3). (B to F) A549 cells were infected with H1N1/H7N9/H9N2 virus at an MOI of 1 for 8 and 16 h, and PBS was used as a negative control. (**B**) M1 and NP vRNAs levels were detected by RT-qPCR (*n* = 3) (top) and visualized by agarose gel electrophoresis (bottom). Data were normalized to *GAPDH* and are presented as mean ± standard deviation (SD). (**C**) Clustered heatmap of 2818 expressed circRNAs in the three virus-infected A549 cells. The relative expression values are represented by color, and increase from blue to red (log scale 2, from −2 to +2). (**D**) Volcano plots illustrating DE circRNAs in H1N1-, H7N9-, or H9N2-infected cells compared with mock-infected cells. The vertical line on the volcano diagram is a 2-fold change threshold, and the horizontal line is the *q*-value < 0.05 threshold. (**E**) Genomic origin and length distribution of differentially up-regulated circRNAs. (**F**) Chromosome distribution and abundance of up-regulated circRNAs.

To identify the difference between AIV and human IAV replication in human cells, we inoculated A549 cells with H1N1, H7N9, or H9N2 virus at an MOI of 0.001, followed by replication kinetics examination. Cell supernatants were harvested at the indicated times post-infection and used for plaque assays to examine viral titers. We found that H7N9 and H9N2 could infect, but their replicative capacity in A549 cells was lower than that of H1N1 virus (Figure 1A). We also verified the expression level of viral NP and M1 transcripts by RT-qPCR, and viral transcripts were also readily identified as strongly up-regulated in the infected cells by agarose gel electrophoresis (Figure 1B). These data confirmed that H7N9 and H9N2 virus can infect and replicate in A549 cells.

### 2.2. Differentially Expressed circRNAs Were Up-Regulated after Infection by AIV

To identify circRNAs involved in AIV replication in A549 cells, we extracted total RNA from A549 cells infected with H1N1, H7N9, or H9N2, at 8 and 16 h post-infection (hpi) and generated circRNA sequencing libraries to perform deep sequencing. The raw sequencing data are available at the NCBI with accession number PRJNA868213. The dataset contains differentially expressed (DE) circRNAs in H1N1-, H7N9-, and H9N2-infected cells at 8 and 16 hpi compared with PBS treatment. We found a total of 10,646 DE circRNAs, and the circRNA expression profiles were then analyzed by hierarchical clustering. Only 475 common significant DE circRNAs were found among the three strains, and they had different regulatory fold changes in each strain. However, these circRNAs were more commonly up-regulated by H7N9 and H9N2 than H1N1 virus (Figure 1C). These results indicate that the three strains induce specific regulatory circRNAs, with avian strains inducing more.

As is shown in Figure 1D, there were 1679, 1753, and 2294 circRNAs significantly up-regulated by H1N1, H7N9, and H9N2, respectively, at 8 hpi, and 2020, 5299, and 3018 circRNAs significantly up-regulated by H1N1, H7N9, and H9N2, respectively, at 16 hpi. This suggests that the total number of all regulated circRNAs increases with the duration of infection in all three strains. In addition, the number of up-regulated circRNAs was greater than that of down-regulated circRNAs in all strains. These results indicate that infection with H7N9 or H9N2 up-regulated more circRNAs than H1N1 virus at both time points, especially at 16 hpi. Most DE circRNAs were generated from exons in the three strains at each time point (Figure 1E). The length of the circRNAs ranged from 150 to 10^5^ nt. At both two time points, most circRNAs were <2500 nt and >200 nt (Figure 1E). We explored the chromosomal distribution and abundance of DE circRNAs based on the source of formation of all detected circRNAs. We found that DE circRNAs were widely distributed among all chromosomes, though the number from each chromosome varied. At both time points, the majority of the altered circRNAs were derived from chromosome 1, with chromosome Y being the least abundant, followed by chromosome 21 (Figure 1F). Altogether, AIV induced more differential up-regulation of features mostly similar to circRNA expression.

### 2.3. Functional Prediction of AIV-Specific Induced DE circRNAs by Their Influence on cis-Target Genes

**Figure 2:**
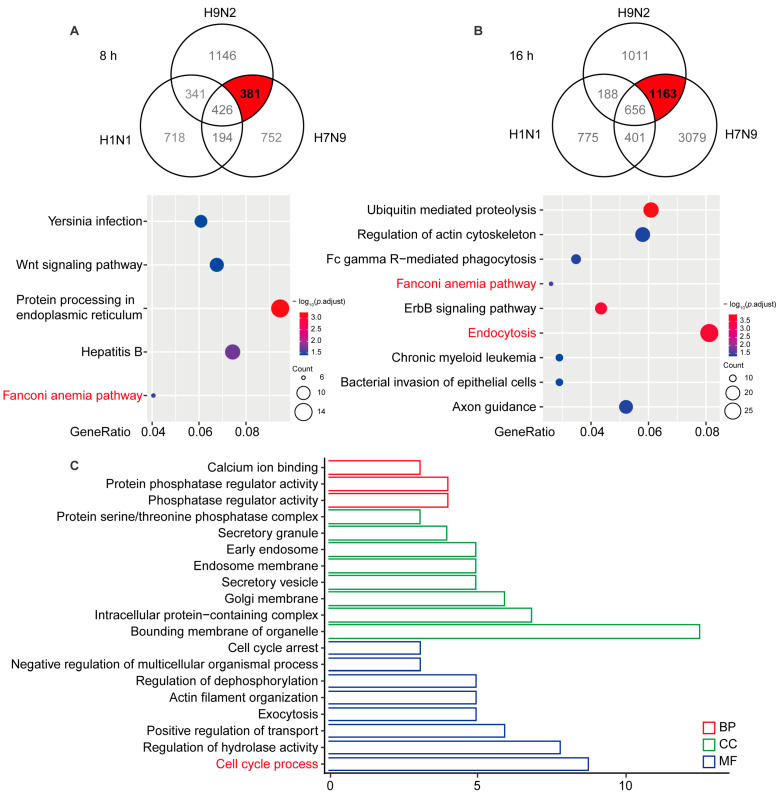
Significantly up-regulated avian virus-specific circRNAs by functional analysis of cis-targeting genes. (**A**) A549 cells infected with H1N1/H7N9/H9N2 subtype IAV for 8 h. The Venn diagram reflects significantly up-regulated circRNAs (top), and the host gene-enriched pathways of circRNAs specifically up-regulated by avian origin IAV were analyzed by KEGG (bottom). (**B**) A549 cells infected with H1N1/H7N9/H9N2 subtype IAV for 16 h. The Venn diagram reflects significantly up-regulated circRNAs (top) and KEGG pathway analysis (bottom). Gene ratio is the ratio of the number of DE genes recorded in a pathway item to the number of all genes recorded in that pathway item. The greater the proportion of genes, the higher the intensity. *p*-values were adjusted ranging from 1.5 to 3.5 (blue to red). Lower *p*-adjustment indicates higher intensity. (**C**) GO analysis of specifically up-regulated host genes of circRNAs at 8 and 16 h. GO enrichment of these genes indicates that biological processes and molecular functions are most enriched, depending on the *p*-value.

To identify AIV-specific DE circRNAs that are induced in viral replication, we compared the circRNA profiles of H1N1-, H7N9-, and H9N2-infected cells. The Venn diagrams shown in Figure 2A,B illustrate 381 and 1163 circRNAs overlapping at 8 and 16 hpi, respectively. To better comprehend AIV-specific DE circRNA up-regulation characteristics, we performed a KEGG analysis of the parental genes of the DE circRNAs, which indicated that the parental genes were enriched in five pathways at 8 hpi and nine pathways at 16 hpi, among which “Fanconi anemia pathway” was enriched at 8 and 16 hpi, “Protein processing in endoplasmic reticulum” was principally enriched at 8 hpi, and “Endocytosis” and “Ubiquitin mediated proteolysis” was principally enriched at 16 hpi. To analyze the biological process in which AIV-specific up-regulated circRNAs may be involved, we overlapped the parental genes at 8 and 16 hpi and found that common genes affecting the GO terms in biological process (BP), cellular component (CC), and molecular function (MF) were enriched in “Protein phosphatase regulator activity”, “Bounding membrane of organelle”, and “Cell cycle process” (Figure 2C). It has been reported that some Fanconi anemia genes interact with influenza viral protein M2, and the Fanconi anemia pathway plays an important role in mitophagy and viral clearance [24,25]. In addition, the “Endocytosis” pathway is important for IAV entry into cells, and cell cycle retention in the G0 phase favors virus replication. These results indicate that AIV-specific up-regulation of circRNAs may at most regulate viral replication through the “Fanconi anemia pathway”, “Endocytosis”, and “Cell cycle process” biological processes.

### 2.4. Validation of Circular Characteristics of Selected circRNAs

**Figure 3:**
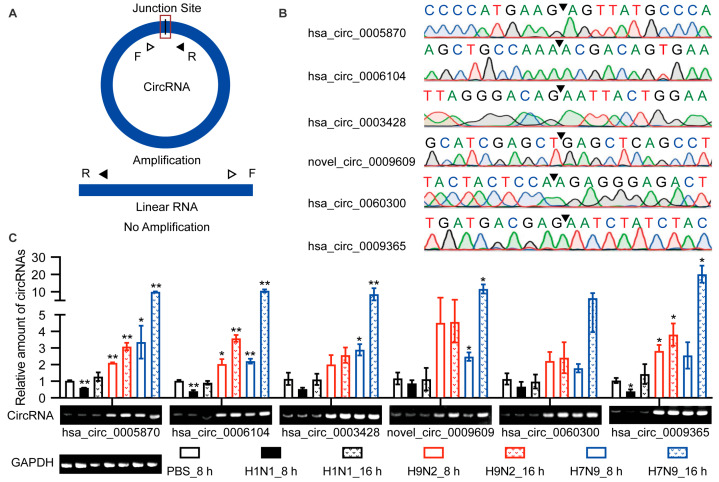
Verification of the circular properties of the selected circRNAs. (**A**) Schematic diagram of PCR primer. The product is expected to be amplified only from circRNAs, not from linear RNAs. (**B**) The total RNAs were treated with RNase R. The junction sites of circRNAs were identified by Sanger sequencing and are represented by arrows. (**C**) Validation of the differential expression of circRNAs during IAV infection. Six circRNAs were selected, quantified by RT-qPCR, and primers were designed to amplify the junction region. Data were normalized to *GAPDH*, and the mean ± SD (*n* = 3) is shown. * *p* < 0.05 and ** *p* < 0.01.

The above identification of circRNAs was based on RNA-seq. However, many factors may lead to false positives for recognition, such as sequence homology, degenerate sequences at exon boundaries, and template switching. Therefore, we selected six up-regulated circRNAs for functional verification based on the KEGG pathway and GO analyses. Hsa_circ_0005870 and hsa_circ_0006104 were among the top 10 specifically up-regulated by AIV and were enriched during GO analysis. Novel_circ_0009609, hsa_circ_0060300, and hsa_circ_0009365 were up-regulated at both time points and were enriched in the endocytosis KEGG pathway. These circRNAs were quantified by RT-qPCR (Figure 3A; Supplemental Table S1). The junction sites were analyzed by Sanger sequencing, and the circRNAs circulation was confirmed (Figure 3B). The differential expression of these six circRNAs was confirmed by RT-qPCR and visualized by agarose gel electrophoresis (Figure 3C). Taken together, differential expression of candidate circRNAs was verified in A549 cells, and their existence was confirmed.

### 2.5. AIV-Specific Up-Regulated circRNAs Regulate AIV Replication

**Figure 4:**
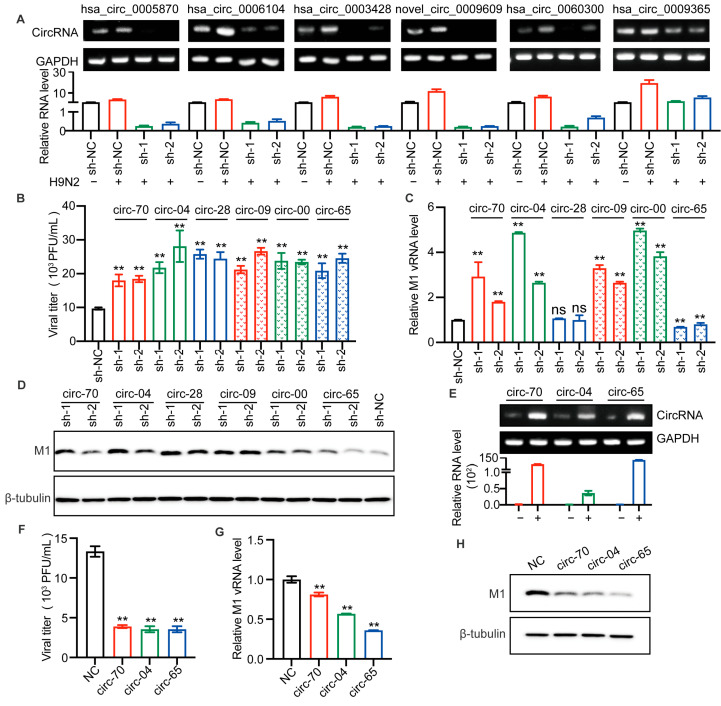
Selected circRNAs affect H9N2 virus replication. (**A**–**D**) The candidate circRNAs in A549 cells were silenced by lentiviral packaged shRNAs for 48 h and then infected with H9N2 virus at 12 h (MOI = 0.5). (**A**) The levels of the six circRNAs were detected by RT-qPCR (*n* = 3) and visualized by agarose gel electrophoresis. Data were normalized to *GAPDH* and expressed as mean ± SD. “−” stands for PBS treatment, and “+” indicates treatment with H9N2 virus. (**B**) The virus titer in supernatants was measured by plaque assays, ** *p* < 0.01. (**C**) The level of AIV M1 vRNA was detected by RT-qPCR (*n* = 3). Data were normalized to *GAPDH* and expressed as mean ± SD. (**D**) Cell lysates were harvested for Western blotting assays using the indicated antibodies. (**E**–**H**) Overexpression of the indicated circRNAs in 293T cells by plasmid transfection for 24 h, followed by H9N2 virus infection at 12 h (MOI = 0.5). (**E**) The overexpression levels of the circRNAs were detected by RT-qPCR (*n* = 3) and visualized by agarose gel electrophoresis. Data were normalized to *GAPDH* and expressed as mean ± SD. “−” stands for PBS treatment, and “+” indicates treatment with H9N2 virus. Cell supernatants were harvested for virus titers (**F**), and for RT-qPCR (*n* = 3) to detect the expression level of viral M1 vRNA (**G**), and the remaining cell lysates were used for immunoblotting for viral M1 protein (**H**). Data were normalized to *GAPDH* and expressed as mean ± SD.

We sought to determine whether the up-regulated circRNAs identified above would affect AIV replication. We utilized lentiviral packaged short hairpin RNA (shRNA) to silence the selected circRNAs. Each circRNA was silenced by two shRNAs covering the junction site. Figure 4A shows the efficiency of circRNA splicing.

It is well known that H9N2 virus has been shown to be the genetic donor for the novel H7N9 and H10N8 AIV, that cause human death. At the same time, serological investigation has shown that humans are more susceptible to H9N2 virus than panzootic H5 and H7 subtype AIVs [26]. Therefore, we were more curious to investigate the effect of circRNAs on H9N2 replication. After lentiviral infection, A549 cells were infected with H9N2 virus, and the supernatants were harvested for viral titer detection. The results suggested that the silencing of all selected circRNAs enhanced H9N2 virus proliferation (Figure 4B). To further confirm the effect of those circRNAs, we detected viral M1 RNA. Interestingly, has_circ_0003428 had no significant effect on M1 vRNA transcription, while has_circ_0009365 significantly down-regulated M1 vRNA transcription. More importantly, the other selected circRNAs all similarly significantly up-regulated vRNA transcription (Figure 4C). In addition, we found that knockdown of circRNAs increased the level of the viral M1 protein (Figure 4D). To verify the antiviral effect of the expression of hsa_circ_0005870 and hsa_circ_0006104 and the function of has_circ_0009365, we constructed three plasmids to overexpress these circRNAs. The levels of the three circRNAs were, respectively, increased in plasmid-transfected 293T cells (Figure 4E). We then compared the replication of H9N2 virus in 293T cells overexpressing circRNAs compared with those without circRNA. The viral titers, M1 vRNA level, and M1 protein level in circRNA-overexpressing cells were lower than in controls (Figure 4F–H). Altogether, we found silencing of selected circRNAs significantly promoted AIV replication in A549 cells, whereas overexpression of circRNAs inhibited AIV replication. These results indicate that the up-regulated circRNAs can act as host barriers to restrict AIV proliferation.

### 2.6. Construction and Extension of circRNA-miRNA-mRNA Networks

**Figure 5:**
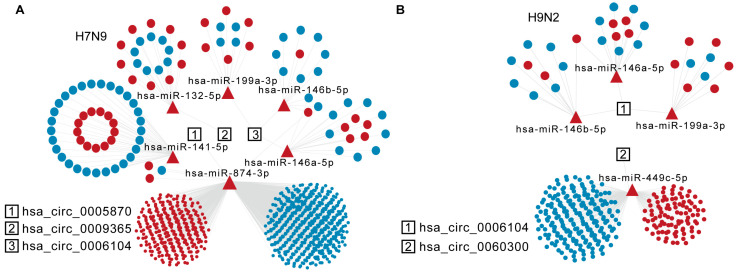
Specific analysis of significantly up-regulated trans-targeted genes of circRNAs. (**A**) The diagram displays significantly up-regulated circRNAs in the H7N9 virus 16 hpi ceRNA network. (**B**) The diagram displays significantly up-regulated circRNAs in the H9N2 virus 16 hpi ceRNA network. The circRNAs, miRNAs, and mRNAs are indicated by rectangles, triangles, and ellipses, respectively.

circRNAs act as different miRNA sponges, regulating mRNAs either in *cis* or in *trans*. We wondered whether specific AIV-up-regulated circRNAs would sponge DE miRNAs and, thus, affect mRNA stability in host cells. The DE miRNAs and DE mRNAs at 8 h and 16 h after H9N2 and H7N9 infection were analyzed by RNA sequencing. Then, miRNA response elements (MREs) and circRNA and miRNA target mRNAs were predicted based on TargetScan (Release 7.2) and miRanda (v3.3a). The detailed statistics for all up-regulated circRNAs and predicted miRNA of H7N9 and H9N2 post infection 16 h are listed in Supplementary Table S2. The ceRNA analysis of the selected circRNAs is shown in Figure 5A,B. Of the six candidate circRNAs, three were involved in the network for H7N9 virus, and two for H9N2 virus at 16 h. Hsa_circ_0006104 is a common circRNA participant in H7N9 and H9N2 virus infections, affecting the common has-miR-146a-5p, has-miR-146b-5p, and has-miR-199a-3p. Hsa_circ_0005870 and hsa_circ_0009365 are genes specific for DE miRNAs uptake in H7N9-infected cells, while hsa_circ_0060300 is only found in the H9N2 virus ceRNA network. Hsa_circ_0005870 could specifically affect a large mRNA in H7N9 virus-infected cells by interacting with has-miR-141-5p and has-miR-874-3p at 16 h. Hsa_circ_0060300 can affect mRNA by absorbing has-miR-449c-5p. Taken together, these results indicate that AIV-specific up-regulated circRNAs may absorb DE miRNA to regulate host mRNA and then restrict AIV replication.

## 3. Discussion

It is well known that most infectious diseases in humans originate from animal reservoirs and understanding how the pathogen can cross the barrier of the host species, or how the host establishes the barrier, is fundamental for control and eradication. Previously, studies related to IAV have focused on antiviral proteins or mRNAs. In recent years, more studies have reported that ncRNAs are involved in IAV replication. However, most focus on a single pathway or protein, especially associated with IAV-regulated circRNAs. Studies explain the mechanism of action of circRNAs and how they promote IAV replication, but large-scale screening has not yet been performed. In this study, we used H7N9, H9N2, and H1N1 subtype viruses as models to verify infection and replication in A549 cells. A large number of DE circRNAs, mRNAs, and some miRNAs were found in IAV replication by RNA-seq analysis. Particularly compared with H1N1 virus, there were many circRNAs specifically up-regulated by AIV. Most of the up-regulated circRNAs were formed from exons, which readily leave the nucleus and enter the cytoplasm. Therefore, they may regulate the transcription of parental genes in the nucleus or act as miRNA sponges to regulate mRNA in the cytoplasm and, thus, participate in AIV replication. The AIV-specific up-regulated circRNAs may be involved in AIV replication through the “Fanconi anemia pathway”, “Endocytosis” pathway, and “Cell cycle process” biological process.

IAV uses the hemagglutinin molecule on the viral coat for attachment and enters cells before it replicates. AIV preferentially binds to sialic acid-α-2,3 (SAα2,3) on the host cell membrane, whereas human-IAV preferentially binds to sialic acid-α-2,6 (SAα2,6) [9]. This difference, coupled with the predominance of sialic acid-α-2,6 on A549 cells [27,28], may explain why we found restricted AIV replication in A549 cells. Interestingly, despite the low replication ability, AIV can induce more circRNAs than human IAV in A549 cells. Regarding the possible mechanisms, we hypothesized that different recognition of SAα2,3- and SAα2,6-binding viruses by receptors on A549 cells could lead to different activation of signaling cascades, leading to AIV induction of more circRNAs, which is independent of viral replication. Previous studies have shown that viruses with preferential affinity for SAα2,3 induce higher levels of ISGs and proinflammatory cytokines in human dendritic cells (DCs) compared with viruses specific for SAα2,6 binding, and that these differences are independent of viral replication since infected viruses are inactivated by UV [29]. Subsequently, they found that DCs and respiratory epithelial cells exhibited higher levels of proinflammatory genes than SAα2,6-affinity viruses after infection with SAα2,3-affinity viruses. DCs and normal human bronchial epithelial cells contain SAα2,3 and SAα2,6 on the surface, similar to A549 cells [30]. We hypothesized that the receptor specificity of AIV might be related to the increased circRNA levels in infected A549 cells. In follow-up work, we will try to use AIV to infect primary normal DCs and human bronchial epithelial cells to verify the transcription levels of six selected circRNAs. Lentiviral overexpression and knockdown of circRNAs to verify their role in regulating AIV infection in these primary cells. In addition, it has been reported that AIV tends to be pathogenic to mice that are not pre-adapted due to the mouse lower respiratory tract containing α-2,3 SA [31,32]. We could overexpress circRNAs using adeno-associated viruses and verify their function in AIV replication in vivo.

Once IAV enters a host cell, the virus must cooperate with host cell processes in order to replicate. AIVs use adaptive mutations in amino acid residues that enable them to cross the species barrier from avians to humans [33]. Notably, our previous study found that some of the highly infectious human H7N9 virus strains used in our experiments have identical poultry residues at sites 627E and 701D of PB2 [18], suggesting that there may be other mechanisms by which AIV highly infects human cells. Recent studies report that IAV-induced circRNAs promote IAV replication [34,35], which is speculated to also occur during AIV replication. However, based on our results, it appears that more circRNAs are involved in the human barriers to inhibiting AIV replication. Interestingly, knockdown of hsa_circ_0003428 and hsa_circ_0009365 could significantly promote the proliferation of H9N2 and up-regulate the level of viral M1 protein, but the transcription level of M1 vRNA was not significantly increased or even decreased. When the virus enters the cell, viral proteins will be modified by host cells. Wang J et al. reported that host lncRNA IPAN promotes IAV replication by regulating viral protein stability [36]. For hsa_circ_0003428, it may affect the stability of H9N2 M1 protein and inhibit the proliferation of H9N2 virus, but does not affect the transcription level of vRNA. For hsa_circ_0009365, it may have multiple functions, such as inhibiting H9N2 budding and vRNA transcription or stability. This may explain that when knockdown of hsa_circ_0009365 promoted AIV budding, more M1 protein and vRNA were assembled into new viral particles and released into the supernatant, whereas inhibition of M1 vRNA transcription or stability by overexpression of hSA_circ_0009365 resulted in reduced H9N2 virus titers. These AIV-specific up-regulated circRNAs might interact with AIV viral proteins or regulate host/viral mRNAs’ stability and translation to participate in AIV replication. Therefore, we further host mRNAs by cis or tans.

When IAV infects cells, a series of signaling pathways are activated. We analyzed circRNA-associated parental genes, and various immune-related genes, such as cytokines, chemokines, and signaling pathway molecules related to a set of biological processes. IAV attaches to human sialic acid receptors and enters cells by endocytosis [37]. Novel_circ_0009609, hsa_circ_0060300, and hsa_circ_0009365 were spliced from the exons of ARFGEF2, SRC, and PRKCZ, respectively, and were enriched in the “Endocytosis” pathway. Hsa_circ_0003428 was formed from POLI, which was enriched in the “Fanconi anemia pathway”. Has_circ_0005870 and has_circ_0006104 were linked back by SETD2 and DCTN2, respectively, and were enriched in the “Cell cycle process”. We verified that all six circRNAs inhibited H9N2 replication. Thus far, few studies related to these circRNA mechanisms have been reported. Only has_circ_0005870 has been shown to be significantly down-regulated in the plasma of hypertensive patients and is also associated with many biological processes, such as cellular responses to stress [38]. Hsa_circ_0060300 is associated with Hepatitis B virus [39], but the mechanisms of these circRNAs have not been reported. Similarly, the mechanisms involved in regulating IAV replication are unknown. It has been reported that IAV infection induces activation of the Src homology region 2-containing protein tyrosine phosphatase 2 (SHP2), which is critical for IAV-induced EGFR/ERK pathway inhibition of the host antiviral response [40]. In addition, SETD2 directly mediates STAT1 methylation on lysine 525 through its methyltransferase activity, thereby enhancing IFN-activated STAT1 phosphorylation and the antiviral cellular response [41]. SETD2 expression is closely correlated with TE induction and specifically interacts with the NSP9 protein of SARS-CoV-2. In IAV-infected A549 cells, there is a global increase in TE subfamily expression in all TE families but not SARS-CoV-2 [42]. In NS1 (RNS1-SD30)-infected cells lacking the eIF4GI-binding domain, DCTN2 expression is up-regulated, and chMDA5 induces enhanced IFN-β promoter activity. We further wonder whether these circRNAs regulate AIV replication by participating in these processes.

At the same time, we identified some specifically up-regulated miRNAs that may be targeted by circRNAs after AIV infection. Among them, has-miR-146a-3p was also reported to be significantly up-regulated in asymptomatic SARS-CoV-2 infected individuals compared with healthy controls [43]. Previous studies have reported that miR-146a inhibits the NF-kB pathway by down-regulating TLR3 and TRAF6, thereby reducing inflammation [44]. Moreover, human infections with H9N2 virus exhibit only mild influenza-like symptoms [45], whereas human infections with H7 are usually mild, causing conjunctivitis or mild respiratory symptoms [46]. Has-miR-146a-3p may be involved in inhibiting exaggerated inflammatory responses in the process of AIV infection. An increasing number of studies report that H7 may be lethal [46,47]. Severe H7N9 virus infection is characterized by high fever and severe respiratory symptoms, which may pose a serious threat to human health, but human pathogenesis is still unclear. Thus, an understanding of the mechanism of AIV cross-species transmission is urgently required.

In conclusion, we demonstrated distinct circRNA expression profiles and bioinformatics analyses in A549 cells infected with LPAIV, HPAIV, or human-IAV, and identified many AIV-specific up-regulated circRNAs that limit IAV replication. We predicted that they may regulate parental genes or act as sponges for miRNAs by affecting the biological processes of “Fanconi anemia pathway”, “Endocytosis” pathway, and “Cell cycle process”. It is necessary to further study the mechanism by which circRNAs restrict AIV replication and screen out the circRNAs that promote AIV replication. This will increase our understanding of the host barrier and how AIV adapts to new hosts.

## 4. Material and Methods

### 4.1. Ethics Statement

All experiments involving the H1N1 subtype IAV (A/WSN/1933, WSN strain) and H9N2 subtype IAV (A/chicken/Hebei/LC/2008(H9N2), HB08 strain) were conducted in a biosafety level 2 (BSL2) laboratory, and the analytical samples and protocols used were approved by the Institute of Microbiology, Chinese Academy of Sciences, Research Ethics Committee (license number: PZIMCAS2021002; approval date: 4 March 2021). All experiments using the H7N9 (A/chicken/Jiangsu/JT186/2017, TJ186 strain) subtype IAV strain were conducted in a biosafety level 3 (BSL3) laboratory approved by the Wuhan Institute of Virology, Chinese Academy of Sciences, Research Ethics Committee.

### 4.2. Viruses, Cells, and Plasmids

WSN was propagated in MDCK cells. TJ186 and HB08 viruses were propagated in 10-day-old specific-pathogen-free (SPF) embryonated chicken eggs purchased from Beijing Vital River Animal Technology Co., Ltd. (Beijing, China) (licensed by Charles River) at 37 °C for 72 h. Human lung adenocarcinoma epithelial cells (A549 cells, CCL-185), Madin–Darby canine kidney cells (MDCK cells, CCL-34), and human embryonic kidney HEK293T cells (293T cells, CRL-3216) were purchased from the American Type Culture Collection (ATCC). The cells were cultured in Dulbecco’s modified Eagle’s medium (DMEM, Gibco, Grand Island, NY, USA), high glucose supplemented with 10% (*v*/*v*) fetal bovine serum (FBS, Gibco, Grand Island, NY, USA), 100 U/mL penicillin, and 100 μg/mL streptomycin (Sigma, St. Louis, MO, USA) at 37 °C in a humidified 5% CO_2_ incubator.

The circRNA-shRNA plasmids were constructed using pSIH-shRNA. Briefly, the shRNA sequence was cloned into the *Bam*HI/*Eco*RI sites of pSIH-shRNA. The shRNA sequences are listed in Supplementary Table S1. The circRNA sequences were cloned in the lentiviral pLC5-CIR vector (Gene-seed, Guangzhou, China) to construct overexpression plasmids. The homologous recombination amplification primers are listed in Supplementary Table S1.

### 4.3. Plaque Assays

MDCK cells were seeded in 12-well plates, cell monolayers were washed with PBS, and then infected with 10-fold serial dilutions of the indicated viruses in serum-free DMEM for 1 h. Then, the cells were washed thrice with PBS and overlaid with modified Eagle’s medium (MEM) containing 1 μg/mL of TPCK-trypsin and 1% agarose. The plates were fixed at 4 °C for 10 min and incubated upside-down at 37 °C in a humidified 5% CO_2_ incubator for 72 h. After that, viral titers were calculated by counting the visible plaques.

### 4.4. RT-PCR and RT-qPCR

Total RNA was extracted from A549 cells with 1 mL TRIzol (Invitrogen, Carlsbad, CA, USA) according to the manufacturer’s instructions and then treated with or without RNase R (1 U/μg). First-strand cDNA was synthesized from 1 μg of total RNA using a cDNA Synthesis SuperMix kit (TransGen Biotech, Beijing, China) with Oligo (dT) primer, Random primer, or vRNA primers according to the supplied protocol. RT-qPCR was performed with TB Green Premix Ex Taq II (TaKaRa, Kusatsu, Japan) according to standard procedures. The expression of *GAPDH* was used as an internal control for normalizing circRNAs. The Ct values were generated from an Applied Biosystems 7500 Real-Time PCR System (Thermo Fisher Scientific, Waltham, MA, USA), and the relative expression was analyzed by the 2^−ΔΔCt^ method. PCR primers are listed in Supplementary Table S1.

### 4.5. Virus Infection and RNA Preparation

A549 cells were infected by IAV (MOI = 1) in DMEM at 37 °C for 1 h. The cells were washed with PBS twice and incubated in DMEM with 0.5 μg/mL TPCK-treated trypsin for 8 h and 16 h in 5% CO_2_ at 37 °C. The A549 cells were infected by IAV (MOI = 0.001) and then the supernatants were collected at the indicated times for the growth curve analysis by plaque assays.

The infected cells were collected for RNA deep sequencing. The quality of RNA samples was determined by analyzing ribosomal RNA band integrity, the quantification of RNA was performed using a Qubit (v3.0, Invitrogen, Carlsbad, CA, USA), and the detection of RNA integrity utilized an Agilent 2100 bioanalyzer (Agilent Technologies, Palo Alto, CA, USA). The rRNA was removed with a Ribo-Zero rRNA Depletion Kit (RiboBio, Guangzhou, China), and linear RNA was removed by treatment with RNase R.

### 4.6. Library Construction and RNA-seq Analysis

To construct the circRNA library, 5 μg total RNA per sample was removed from rRNA and linear RNA. Then, the fragmented RNAs were reverse transcribed into cDNA using random primers. The cDNAs were purified, end-repaired, polyadenylated, and ligated with Illumina sequencing adapters. AMPure XP system (Beckman Coulter, Pasadena, CA, USA) was used to select 250- to 300-bp cDNA fragments. Then, uracil-N-glycosylase was used to digest the second strand of cDNA. The digested products were amplified by PCR and clustered by Novogene Biotechnology Co. (Beijing, China) using TruSeq PE Cluster Kit on a cBot Cluster Generation System (v3-cBot-Hs). After cluster generation, the libraries were sequenced on the Illumina platform and 150 bp paired-end reads were generated. To construct the miRNA library, the total RNA was directly added to 3′ and 5′ adapters for ligating the two sides of the miRNA. Then, the miRNAs were reverse transcribed, amplified, and clustered by Novogene Biotechnology Co. on a cBot Cluster Generation System using the TruSeq SR Cluster Kit v3-cBot-HS (Illumina, San Diego, CA, USA), and then, the libraries were sequenced on an Illumina SE50 platform as 50-bp single-end reads. To construct the mRNA library, rRNA was removed from the total RNA, and the fragmented RNAs were reverse transcribed into cDNA by using an oligo (dT) primer. The products were amplified by PCR and sequenced by Novogene Biotechnology Co. (Beijing, China) using Illumina PE150 sequencing.

Reads that contained adapter sequences and low-quality reads were removed. The remaining reads of the circRNA library were mapped with Hisat2 (v2.0.5, Hisat2 Team Maryland, USA), and then, circRNAs were identified by CIRI software (v2.0.5, CIRI Team, Beijing, China). The remaining junction reads were aligned to human circRNAs in the circBase database (http://www.circbase.org/, accessed on 18 January 2021). Clean reads of the miRNA library were screened, and 21 to 22 nt miRNAs were selected for subsequent mapping using Bowtie2 software (v2.0.6, Bowtie2 Team, Maryland, USA) and aligned to miRbase (https://mirbase.org/, accessed on 25 January 2021). Clean reads of the mRNA library were spliced with Stringtie software (v1.3.3, StringTie Team, Maryland, USA) and aligned with Cuffcompare software. DE circRNAs, miRNAs, and mRNAs in each dataset were investigated according to the DEGSeq analysis with *p*-value < 0.05 and |log2FC| > 1 as the threshold. The significantly DE circRNAs, DE miRNAs, and DE mRNAs were investigated by the DESeq2 R package (R-3.1.2). To obtain an overview of the expression profiles of circRNAs, volcano plotting, heat mapping, and chromosome mapping were performed using ggplot 2, heatmap, and karyoploteR, respectively.

### 4.7. GO Enrichment and KEGG Pathway Analysis

GO enrichment analysis for host genes of DE circRNAs was implemented by the topGO R package (Release 2.12). GO terms with corrected *p*-value < 0.05 were considered significantly enriched by differentially expressed genes. KEGG is a database resource for understanding high-level functions and utilities of a biological system, such as the cell, the organism, and the ecosystem, from molecular-level information, especially large-scale molecular datasets generated by genome sequencing and other high-throughput experimental technologies (http://www.genome.jp/kegg/, accessed on 29 May 2022). We used KOBAS software (v2.0, kobas.cbi.pku.edu.cn, Beijing, China) via a hypergeometric with a corrected *p*-value < 0.05. *q*-value is used as a statistical method for estimating the false discovery rate (FDR), which is a conventional significance measure in the analysis of genome-wide expression data, with a corrected *q*-value < 0.05.

### 4.8. Cell Transfection and Generation of Stable Cell Lines

Transfection of the circRNAs overexpression pLC5-ciR vector was conducted by Lipofectamine 2000 (Thermo Fisher Scientific) for 24 h according to the manufacturer’s protocol. Stable knockdown cell lines were generated by transfecting 293T cells with the lentiviral packaging vectors (pRSV, pVSVG, and pMDIG) and pSIH-shRNA for 48 h. Then, the virus-containing supernatants were collected and used to infect A549 cells.

### 4.9. Agarose Gel Electrophoresis and Immunoblotting

The 2% agarose gel was prepared by dissolving 2 g of agarose in 100 mL TAE flow buffer (0.04 mol/L Tris-acetic acid, 0.001 mol/L EDTA) and adding ethidium bromide. The amplification products were mixed with loaded dye (New England Biolabs), loaded on the gel and run at 150 V for 20 min. The amplification products were detected by UV absorbance, and the target stripes were collected for Sanger sequencing. The cells were seeded in 12-well plates in lysis buffer supplemented with protease inhibitor cocktail and RNase inhibitor (20 mmol/L HEPES, pH 7.4, 150 mmol/L NaCl, 10% glycerin, 1% Triton X-100, and 1 mmol/L EDTA). Protein separation was mixed with 5 × SDS-PAGE loading buffer (250 mmol/L Tris-HCL, 10% *w*/*v* SDS, 0.5% BPB, 50% glycerol, 5% 2-Mercaptoethanol), separated by 10% SDS-PAGE gel, and transferred to PVDF membrane. Immunoblotting was visualized with ECL substrate (Tanon, Shanghai, China.).

### 4.10. microRNA Target Site and circRNA-miRNA-mRNA Network Analysis

Based on the ceRNA theory, we constructed ceRNA regulatory networks for the DE circRNAs and DE mRNAs to determine the regulatory relationships among circRNA miRNA, and mRNA. miRanda (http://www.microrna.org/microrna, accessed on 12 March 2022) was used to predict miRNA binding seed sequence sites, and the ceRNA network, which consisted of circRNA-miRNA pairs, and miRNA-mRNA pairs with the same miRNA nodes, were visualized by Cytoscape 3.9.1 (Cytoscape Team, Seattle, WA, USA, https://cytoscape.org, accessed on 12 March 2022).

### 4.11. Statistical Analysis

Data are presented as mean ± standard deviation (SD) from at least three independent experiments unless otherwise indicated. Comparisons between two groups were performed using the two-tailed Student’s *t*-test. *p* < 0.05 was considered significant, with * *p* < 0.05 or ** *p* < 0.01.

## Data Availability

All relevant data were included in this published article. The datasets used and/or analyzed during the current study are available from the corresponding author on request. The raw data for the circRNA, miRNA, and mRNA sequencing were deposited in the NCBI Sequence Read Archives (SRA) database (accession number: PRJNA868213).

## Supplementary Materials

The following are available online at https://www.mdpi.com/article/10.3390/ijms23179901/s1.
